# Knocking Down Gm16685 Decreases Liver Granuloma in Murine Schistosomiasis Japonica

**DOI:** 10.3390/microorganisms11030796

**Published:** 2023-03-21

**Authors:** Ruyu Zhao, Xiaoxue Tang, Huiyao Lin, Chen Xing, Na Xu, Bingxin Dai, Pingping Wang, Wei Shao, Miao Liu, Jijia Shen, Shengqun Deng, Cuiping Ren

**Affiliations:** Department of Microbiology and Parasitology, Anhui Provincial Laboratory of Pathogen Biology, Anhui Key Laboratory of Zoonosis of High Institution, Laboratory of Tropical and Parasitic Diseases Control, School of Basic Medical Sciences, Anhui Medical University, Hefei 230032, China

**Keywords:** *Schistosoma japonicum*, long noncoding RNAs, liver granuloma, macrophage polarization

## Abstract

Long noncoding RNAs (lncRNAs) can regulate key genes and pathways in liver disease development. Moreover, macrophages are speculated to play an important role in regulating granulomatous inflammation during schistosomiasis. However, the role of lncRNAs in the formation of liver granulomas by influencing the polarization of macrophages in *Schistosoma japonicum* infection is unclear. Our study aimed to determine whether lncRNAs can play a role in *S. japonicum*-induced hepatic egg granulomas and elucidate their effect on macrophages. We established *S. japonicum* infection models and screened the target lncRNA Gm16685 highly expressed in schistosomiasis mice using high-throughput sequencing. Hematoxylin and eosin staining revealed that the knockdown of Gm16685 reduced the area of egg granulomas. Moreover, M1 macrophage factor genes were significantly downregulated in Gm16685 knockdown livers. Meanwhile, M2 macrophage factor genes were significantly upregulated, which was consistent with the protein detection results. Hepatocytes, hepatic stellate cells, and macrophages were isolated from mouse models infected with *S. japonicum*, with Gm16685 being significantly upregulated in macrophages. Moreover, the knockdown of Gm16685 in RAW264.7 cells revealed similar results to in liver tissue. RNA fluorescence *in situ* hybridization (FISH) and nucleocytoplasmic separation experiments revealed that Gm16685 was predominantly localized in the cytoplasm of cells. We found that miR-205-5p was upregulated after Gm16685 was knocked down. After overexpression of miR-205-5p, the expression of Gm16685 and inflammatory factors was significantly downregulated. These results indicate that Gm16685 can participate in the pathogenesis of hepatic disease in schistosomiasis and promote M1 macrophage polarization by regulating miR-205-5p. Thus, our study may provide a new target for schistosomiasis japonica treatment.

## 1. Introduction

Schistosomiasis is an important but often neglected tropical disease that poses a serious threat to the health of more than 250 million people worldwide [1,2,3]. Schistosomiasis is mainly caused by *Schistosoma hematobium*, *S. mansoni*, and *S. japonicum* [4,5]. As the pathogen of hepatointestinal schistosomiasis, *S. japonicum* is primarily prevalent in China, with a low prevalence in the Philippines and Indonesia [4,5]. Female and male *S. japonicum* worms parasitize human veins and mate to produce fertilized eggs [6]. These eggs secrete antigenic glycoproteins, which promote their transfer from blood vessels (spawning sites) into the intestinal cavity or bladder by inducing inflammatory reactions. Moreover, these soluble egg antigens (SEAs) can also induce granulomas, which are collections of inflammatory cells, around the eggs and in the surrounding tissues [7]. The main cellular components of granulomas include macrophages, which play a significant role in egg granulomatous inflammation caused by *S. japonicum* [8,9,10]. Macrophages are divided into two types: M1 macrophages and M2 macrophages, which have opposite functions in the inflammatory response [11]. M1 macrophages promote the progression of inflammatory responses by secreting proinflammatory cytokines such as IL-1β, whereas M2 macrophages exhibit anti-inflammatory effects by inducing the high expression of IL-10 and TGFβ [12,13]. Additionally, macrophages can govern inflammation caused by schistosomiasis [14]. However, the regulatory mechanism of macrophage polarization remains unclear.

Long noncoding RNAs (lncRNAs) are defined as a group of RNA transcripts that are longer than 200 nucleotides without coding potential [15,16]. They may be in the cytoplasm or nucleus and play a crucial role in cellular processes [17]. Notably, lncRNA is an essential potential regulatory molecule of tumor cells, playing a pivotal role in tumor metastasis, immune escape, metabolism, and angiogenesis, becoming a core in tumor-related signaling pathways [18]. Increasing evidence also suggests that the aberrant expression of lncRNAs is closely related to the initiation and progression of viral infection [19], cancer [20], liver disease, and other diseases [21]. Currently, lncRNA-related research in the liver mainly focuses on nonalcoholic steatohepatitis, hepatocellular carcinoma, and cholestatic liver disease [22,23,24]. RNA sequencing analysis revealed the differential expression of many lncRNAs in the liver after 25 days of infection with *S. japonicum*, suggesting that lncRNAs could participate in the pathogenesis of the liver after infection with *S. japonicum*. [25].

MicroRNA (miRNA) is a small noncoding RNA molecule that can target mRNA and regulate translation inhibition. miRNA participates in many biological processes and can also induce messenger RNA (mRNA) degradation or prevent its translation [26]. lncRNA can play its biological function as competitive endogenous RNA (ceRNA), thus reducing the inhibition of miRNA-mediated downstream transcripts of miRNA [27].

However, it is unclear how the expression profile and functions of these lncRNAs change during schistosomiasis. This study aims to establish the expression profile of lncRNAs in the liver of *S. japonicum*-infected mice at various time points and explore the potential regulatory mechanism of lncRNAs in schistosomiasis.

## 2. Materials and Methods

### 2.1. Schistosomiasis Mouse Model

Six-week-old female C57BL/6 mice were acquired from the Animal Center of Anhui Medical University and housed in a special pathogen-free animal room. Animal-related experiments were conducted according to the regulations on management and animal ethical standards. Mice were assigned into four groups stochastically, with 6 mice in each group. For the *S. japonicum* infection mouse model, the abdominal hair of mice was removed, and three groups of mice were selected to infect 18 ± 2 *S. cercariae*. The key period of liver granuloma formation is 45 days after schistosomiasis infection [28]. Therefore, after 45 days of infection with *S. japonicum*, the mice were divided into the model, LV-shRNA, and LV-sh-Gm16685 groups, and the corresponding lentivirus was injected into the tail vein of mice in the LV-shRNA and LV-sh-Gm16685 groups. Three weeks later, the mice were dissected for further experimentation.

### 2.2. Cell Culture

RAW 264.7 cells were maintained in DMEM (Gibco, New York, NY, USA) supplemented with 10% fetal bovine serum at 5% CO_2_ and 37 °C. For lipopolysaccharide (LPS) stimulation, the cells were placed into a six-well plate and incubated overnight. Subsequently, diluted LPS was added, and the final concentration was 2 ng/mL. Then, the cells were incubated for 12 h.

### 2.3. Quantitative Reverse Transcription-PCR (qRT-PCR)

Total RNA from RAW264.7 cells or liver tissues was extracted using TRIzol (Ambion, Austin, TX, USA). According to the manufacturer’s instructions, extracted RNA was reverse transcribed with Evo M-MLV RT Premix. Subsequently, the SYBR**^®^** Green Premix Pro Taq HS qRT-PCR Kit (Accurate Biotechnology Co., Ltd., Changsha, China) was used for subsequent qRT-PCR experiments. Actin was chosen as an internal control for the experiments, and gene expression was computed using the 2^−ΔΔCt^ method. The primer sequences are displayed in Table S1.

### 2.4. Cell Transfection

To silence and overexpress Gm16685, sh-Gm16685, Gm16685-expressing plasmid, and the negative control (sh-NC) were synthesized by Wuhan Miaoling Biotechnology Co., Ltd. (Wuhan, China). miR-205-5p mimics were synthesized by General Biol Co., Ltd. (Chuzhou, China). For transfection, cells were placed in a 6-well culture plate and cultured overnight until 70–80% confluence was reached. EndoFectin™ MAX transfection reagent (GeneCopoeia, Maryland, NY, USA) was also used for transfection according to the instructions. After thirty-six hours, RAW264.7 cells were subjected to treatment with LPS for 12 h. Finally, transfection efficiency was detected.

### 2.5. Histological Examination

The liver tissues were washed with phosphate-buffered saline (PBS) and then placed in 4% paraformaldehyde. Then, 4 µm thick paraffin slices were obtained. The pathological changes in the liver tissues were observed using hematoxylin and eosin (H&E) staining. The proportion of egg granuloma area in the total area of the H&E slice was determined using ImageJ-win64.

### 2.6. Adult Counts and Egg Counts

The adults were extracted from the hepatic portal vein and mesentery of the mice infected with *S. japonicum* for counting. Next, 2 mL of 5% KOH was added to a 5 mL Eppendorf (EP) tube. Then, 0.2 g of mouse liver tissue was separated and cut into pieces in an EP tube. This was incubated at 37 °C for 3 h. Then, 50 µL was isolated from the liquid to count the eggs of *S. japonicum* under the microscope. Counting was performed three times, and the average value was considered. Finally, the total number of eggs of each mouse was calculated.

### 2.7. Isolation of Cells

After anesthesia, the mouse liver tissues were digested with Streptomyces griseus (Sigma, Saint Louis, MO, USA) and collagenase D (Sigma, USA). The digested liver was cut into pieces and then filtered through a sieve. The filtered cell fluid was centrifuged for 5 min to obtain hepatocyte sediment. Then, we centrifuged the supernatant obtained in the previous step at 600× *g* for 10 min, and then took the sediment. We added 5 mL of 20% Nycodenz into the centrifuge tube, mixed it with the sediment, and carefully added 5 mL of 12% Nycodenz and 3mL DMEM on the suspension surface. Then it underwent 1400× *g* centrifugation for 17 min. After centrifugation, the liquid in the centrifuge tube was in a stratified state. One layer of cells in the middle layer was hepatic stellate cells [29].

Other mice were selected to isolate macrophages. The mice were killed and then soaked in 75% alcohol for 5 min. The abdomen was lifted with tweezers, and a small wound was inflicted. To this end, 8 mL PBS was injected, and the peritoneal fluid was removed using a pasteurized straw. Finally, centrifugation was performed to obtain peritoneal macrophage precipitation.

### 2.8. RNA Fluorescence in Situ Hybridization (FISH)

Gm16685 probes were synthesized by Shanghai GenePharma Co., Ltd. (Shanghai, China). Then, RAW264.7 cells were resuspended in complete medium and mixed evenly. The cells were counted, and then the diluted cell suspension was added to the hole where the climbing tablets were placed. The paved 24-well plates were then incubated overnight. Then, FISH was performed following the instructions of Shanghai GenePharma Co., Ltd. Finally, the cells were observed under a 63 × oil lens of a laser confocal microscope.

### 2.9. Nucleocytoplasmic Separation

The NE-PER Nuclear Cytoplasmic Extraction Reagent kit (Thermo Scientific, USA) was used for nucleocytoplasmic separation. RAW264.7 cells were washed and then placed in a 1.5 mL EP tube and centrifuged at 500× *g*. Precooled 500 μL cytoplasmic extraction reagent I was then added to the cell precipitate and vortexed for 15 s to suspend the cell precipitate. This was placed on ice for 10 min. Then, 11 mL of a second cytoplasmic extraction reagent II was added to the sample. It was then placed on ice and centrifuged for 5 min at 16,000× *g*. The supernatant was placed into a new precooled EP tube. Precooled 250 μL NER (Nuclear Extraction Agent) was used to resuspend the sediment (which contained crude nuclei), and then the sample was incubated on ice after vortexing for 15 s. After centrifuging at 4 °C for 10 min, the supernatant (nuclear extract) was transferred into a new precooled EP tube. The RNA of the nuclear extract and cytoplasmic extract were extracted, and the expression of Gm16685 in the nucleus and cytoplasm was further analyzed using qRT-PCR.

### 2.10. Protein Determination Method

Approximately 0.1 g of the same liver part of the three groups was isolated. The liver tissues were homogenized on ice using RIPA buffer (Beyotime Inst. Biotech, Shanghai, China), which was supplemented with phenylmethylsulfonyl fluoride (PMSF), and then centrifuged at 4 °C for 15 min at 12,000 rpm. The supernatant, after cell transfection, was collected and stored. The protein concentrations of liver tissue were detected using the enhanced BCA protein assay kit (Beyotime Inst. Biotech, Shanghai, China). M1 and M2 cytokine determination was performed by Shanghai Universal Biotech Co., Ltd. (Shanghai, China). Murine IL-1β, IL-12A, IL-4, and IL-10 were analyzed using Luminex technology and reagents (R&D Systems, Minneapolis, MN, USA).

### 2.11. Luciferase Reporter Assay

The wild-type (wt) and mutant-type (mut) sequence fragments of Gm16685 (containing the binding sites of miR-205-5p or the mutated binding sites of miR-205-5p) were cloned into the GV272 vector (Shanghai Genechem, Shanghai, China). In 293T cells, the vectors were transfected with miR-205-5p or miR-NC. After 48 h, the activity of luciferase in cells was determined.

### 2.12. miRDB

The NCBI gene database (https://www.ncbi.nlm.nih.gov/, accessed on 1 March 2023) was used to query the sequence of lncRNA, accessed on 1 September 2020. The miRDB database (http://mirdb.org, accessed on 1 March 2023) was used to predict the miRNAs of lncRNA interaction, accessed on 1 March 2022. The lncRNA sequence was used for prediction. The specific binding sites can be viewed through the “Details” on the left side of each result.

### 2.13. Statistical Analysis

GraphPad Prism 8 was used to analyze the data. Data from at least three biological replicates are expressed as the mean ± standard deviation. The difference between the two groups was evaluated using Student’s *t*-test, and *p* < 0.05 was considered statistically significant.

## 3. Results

### 3.1. LncRNA Expression in the Liver of Mice with Schistosomiasis

**Figure 1:**
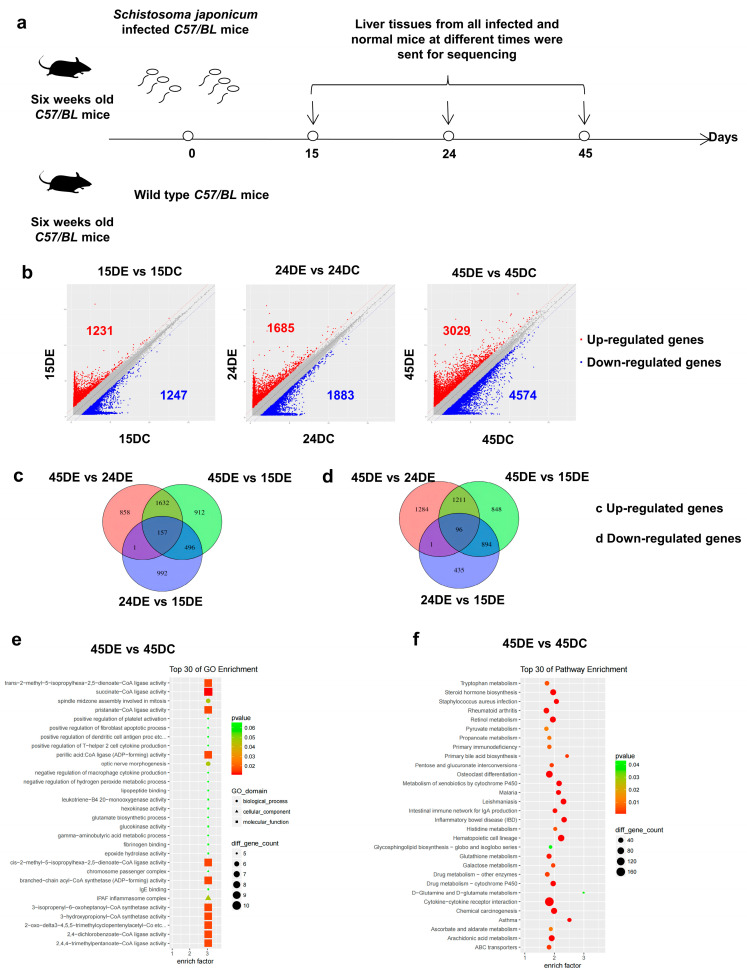
Different expression profiles of lncRNAs in mice at 15, 24, and 45 days after infection with *Schistosoma japonicum*: (**a**) The schematic timeline shows that mice were infected with *S. japonicum*, and the infected mice were sacrificed together with the normal mice on the 15th, 24th, and 45th days. (**b**) Volcano plots showing differentially expressed lncRNAs in liver tissues of the mice on the 15th, 24th, and 45th days after *S. japonicum* infection. (**c**,**d**) The pie chart shows the number of upregulated and downregulated lncRNAs in the liver tissues of mice infected with *S. japonicum* for 15, 24, and 45 days. (15DE, 24DE, and 45DE represent 15, 24, and 45 days after *S. japonicum* infection; 15DC, 24DC, and 45DC denote normal mice raised simultaneously). (**e**,**f**) Gene Ontology and Kyoto Encyclopedia of Genes and Genomes pathway analyses were used to analyze differentially expressed lncRNAs.

To explore the characteristics of lncRNAs in the livers of mice infected with Schistosoma at different time points, *S. japonicum*-infected livers and normal livers were collected and sent to Shanghai Biotechnology Corporation for lncRNA expression profiling (Figure 1a). Compared with normal mice at the same time point, 1231 lncRNAs were upregulated and 1247 lncRNAs were downregulated in the livers of mice infected for 15 days, while 1685 lncRNAs were upregulated and 1883 lncRNAs were downregulated in those infected for 24 days. Moreover, 3029 lncRNAs were upregulated and 4574 lncRNAs were downregulated in mice infected for 45 days (Figure 1b, *p* < 0.05, fold-change > 2). A total of 157 lncRNAs were upregulated and 96 lncRNAs were downregulated in the liver tissues of mice infected with S. japonicum for 15, 24, and 45 days (Figure 1c,d, *p* < 0.05, fold-change > 2). Gene ontology enrichment analysis revealed that at 45 days after infection, the differentially expressed lncRNAs were mainly involved in the biological process of the assembly of the spindle body during mitosis and the morphogenesis of the optic nerve. Furthermore, differentially expressed genes were enriched in the cellular components of the IPAF inflammatory body complex, whereas in terms of molecular function, the genes were highly enriched in coenzyme A ligase activity (Figure 1e). Kyoto Encyclopedia of Genes and Genomes analysis showed that the differentially expressed lncRNAs 45 days after infection were mainly involved in the interaction between cytokines and cytokine receptors, the formation of hematopoietic cells, the differentiation and development of osteoclasts, the biosynthesis of steroid hormones, the metabolism of cytochrome P450, and the metabolism of arachidonic acid (Figure 1f).

### 3.2. The Expression of lncRNA-Gm16685 Was Significantly Increased in the Livers of Mice with Schistosomiasis

**Figure 2:**
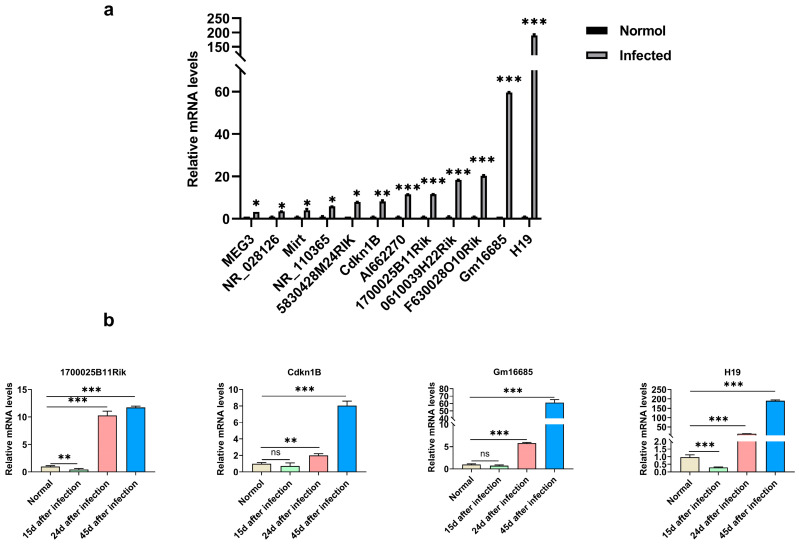
Validation of relative lncRNA expression using quantitative real-time PCR (qRT-PCR): (**a**) The differentially expressed lncRNAs in the liver tissues of model mice (mice infected with *Schistosoma japonicum* for 45 days) and normal mice were verified using qRT-PCR. The relative expression level of lncRNA was consistent with the high-throughput sequencing results. (**b**) The mRNA levels of 1700025B11Rik, Cdkn1B, Gm16685, and H19 in the liver tissues of the mice infected with *S. japonicum* for 15, 24, and 45 days and the liver tissues of the normal group at the same time point were detected using qRT-PCR. Data are presented as the mean ± standard deviation, n = 3. ns, not significant, * *p* < 0.05, ** *p* < 0.01, *** *p* < 0.001.

A total of 12 lncRNAs that were differentially expressed in mouse livers after 45 days of *S. japonicum* infection were identified. These lncRNAs were related or unknown to inflammation, and there are more than two exons in the sequence. They were further verified using qRT-PCR. The results were consistent with the high-throughput sequencing analysis, indicating the feasibility of the screening method (Figure 2a).

Three lncRNAs with unknown function (1700025B11Rik, Cdkn1B) and H19 with the highest differential expression were further selected for qRT-PCR analysis to detect their expression levels in the livers of mice 15, 24, and 45 days after *S. japonicum* infection. The results showed that the expression level of lncRNA-Gm16685 was increased 6-fold (*t*_(4)_ = 37.03, *p* < 0.0001) and 61-fold (*t*_(4)_ = 26.18, *p* < 0.001) after 24 and 45 days of infection, respectively (Figure 2b). Combining the results of high-throughput sequencing and qRT-PCR revealed that Gm16685 was significantly increased in the liver of mice infected with *S. japonicum* after 45 days, except for H19. Therefore, we speculate that Gm16685 could be involved in the progression of schistosomiasis.

### 3.3. Knockdown of Gm16685 Alleviates S. japonicum-Induced Hepatic Granulomas

**Figure 3:**
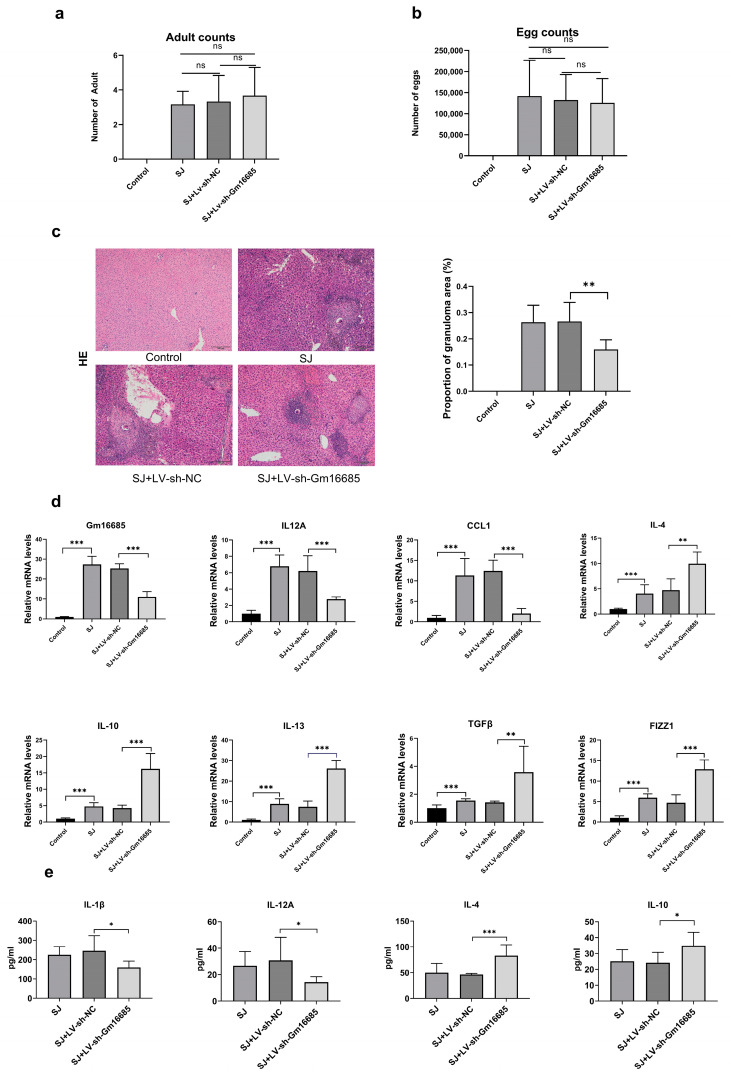
Functional analysis *in vivo*: (**a**) Number of *Schistosoma japonicum* eggs in the liver tissue of the three groups. (**b**) Number of *S. japonicum* adults in the liver tissues of the three groups. (**c**) Hematoxylin and eosin staining was performed to measure the pathological changes in liver tissues (original magnification × 100, scale bar = 200 µm). The area of egg granulomas of *S. japonicum* in the total area of the liver was counted. (**d**) The expression levels of Gm16685, IL-12A, CCL1, IL-4, IL-10, IL-13, TGFβ, and FIZZ1 mRNAs were detected using qRT-PCR in mouse tissues of each group. Control: normal mice; SJ: mice infected with *S. japonicum* for 45 days; SJ+LV-NC: model mice treated with blank vector; SJ+LV-Gm16685: model mice treated with lentivirus that can silence target gene expression. (**e**) Cytokine analysis: Murine IL-1β, IL-12A, IL-4, and IL-10 were analyzed using Luminex technology and reagents in mouse tissues of each group. Data are presented as the mean ± standard deviation, n = 6 mice per group. Ns, not significant, * *p* < 0.05, ** *p* < 0.01, *** *p* < 0.001.

To investigate the potential impact of Gm16685 on egg-induced liver granuloma formation in schistosomiasis, an *S. japonicum* infection model in mice was constructed. Hepatic granuloma areas were significantly decreased (*t*_(10)_ = 3.19, *p* = 0.005) in the Gm16685 knockdown groups. However, there was no significant difference in the parasite and egg count in each group (Figure 3a–c). H&E staining revealed that egg granuloma reaction, inflammatory cell infiltration, and hepatocyte necrosis were alleviated in the Gm16685 knockdown group (Figure 3c). Compared with the control group, M1 macrophage factor genes, including IL-12A (*t*_(10)_ = 4.53, *p <* 0.001) and CCL-1 (*t*_(10)_ = 8.92, *p <* 0.001), were significantly downregulated in Gm16685 knockdown livers. Meanwhile, M2 macrophage factor genes, including IL-4 (*t*_(10)_ = 3.99, *p* = 0.0012), IL-10 (*t*_(10)_ = 6.14, *p* < 0.001), IL-13 (*t*_(10)_ = 9.55, *p <* 0.001), TGF-β (*t*_(10)_ = 2.86, *p* = 0.008), and Fizzl (*t*_(10)_ = 6.74, *p <* 0.001), were significantly upregulated (Figure 3d). Moreover, IL-12A (*t*_(10)_ = 2.26, *p* = 0.023) and IL-1β (*t*_(10)_ = 2.51, *p* = 0.015) protein expression levels significantly decreased, while IL-4 (*t*_(10)_ = 4.31, *p* < 0.001) and IL-10 (*t*_(10)_ = 2.44, *p* = 0.017) protein levels significantly increased in the treatment group (Figure 3e).

### 3.4. Knockdown of Gm16685 Promotes the Polarization of Macrophages to the M2 Phenotype In Vitro

**Figure 4:**
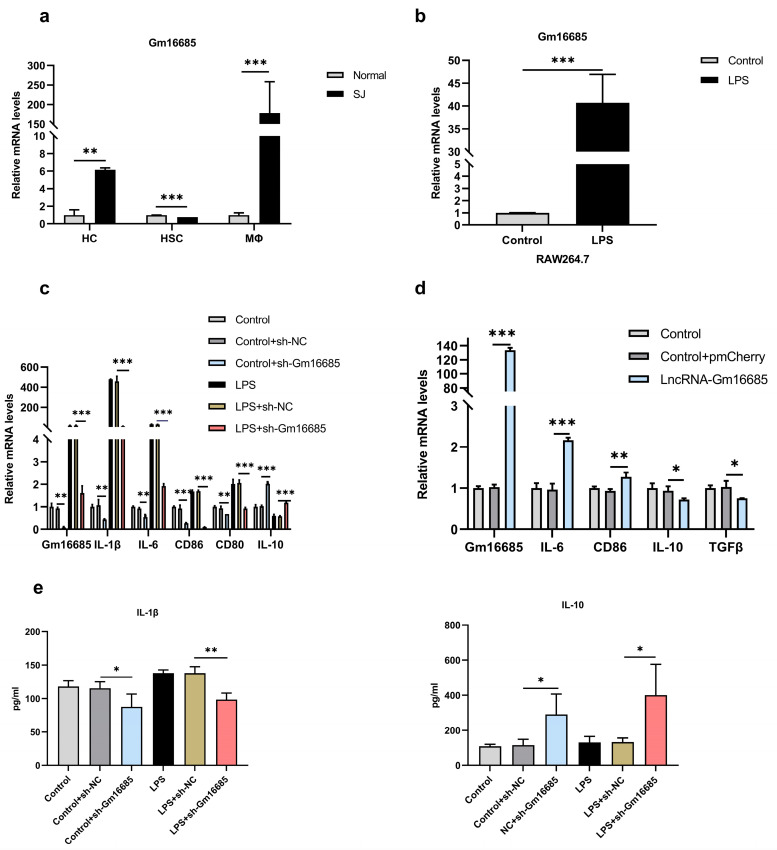
Functional analysis *in vitro*: (**a**) The expression of Gm16685 mRNAs in three primary cells of mice infected with *Schistosoma japonicum* for 45 days. (**b**) The expression of Gm16685 mRNAs in RAW264.7 cells stimulated with lipopolysaccharide was detected using quantitative real-time PCR. (**c**) Knockdown of lncRNA-Gm16685 in RAW 264.7 cells. The expression levels of Gm16685, IL-1β, CD80, CD86, IL-6, and IL-10 mRNAs were detected using qRT-PCR in RAW264.7 cells. (**d**) Overexpression of lncRNA-Gm16685 in RAW 264.7 cells. The expression levels of Gm16685, IL-6, CD86, IL-10, and TGFβ mRNAs were detected using qRT-PCR in RAW264.7 cells. (**e**) Cytokine analysis: Cell supernatant IL-1β and IL-10 were analyzed using Luminex technology and reagents. Data are presented as the mean ± standard deviation, n = 3. ns, not significant, * *p* < 0.05, ** *p* < 0.01, *** *p* < 0.001.

Hepatocytes, hepatic stellate cells, and macrophages were isolated from *S. japonicum*-infected mice and healthy mice. Notably, Gm16685 was highly expressed in activated macrophages (*t*_(4)_ = 3.82, *p* = 0.009) (Figure 4a). Additionally, Gm16685 was significantly upregulated in the activated RAW264.7 cell line (*t*_(4)_ = 11.03, *p* < 0.001) (Figure 4b).

To further evaluate the role of Gm16685 in regulating macrophage polarization, Gm16685 was knocked down with Gm16685-shRNAs in RAW264.7 cells and then treated with LPS or without LPS. The knockdown of Gm16685 decreased the expression levels of IL-1β (*t_(_*_4)_ = 4.22, *p* = 0.007), IL-6 (*t*_(4)_ = 6.20, *p* = 0.002), CD86 (*t*_(4)_ = 7.39, *p* = 0.0008), and CD80 (*t*_(4)_ = 5.02, *p* = 0.004) compared with the control cells, while a higher level of IL-10 (*t*_(4)_ = 29.64, *p <* 0.001) was observed. Moreover, the downregulation of Gm16685 blocked the LPS-induced upregulation of these M1-phenotype genes and reversed the downregulation of M2-phenotype genes in RAW264.7 cells (Figure 4c). However, the overexpression of Gm16685 increased M1-phenotype gene expression (such as IL-6 (*t*_(4)_ = 13. 01, *p* = 0.0001) and CD86 (*t*_(4)_ = 4.84, *p* = 0.004)) and decreased M2-phenotype gene expression (Figure 4d). Simultaneously, treatment with shRNA-Gm16685 caused significant increases in IL-10 (LPS, *t*_(4)_ = 2.63, *p* = 0.029; control, *t*_(4)_ = 2.48, *p* = 0.033) in the supernatant of the cell cultures. In contrast, IL-1β (LPS, *t*_(4)_ = 4.99, *p* = 0.004; control, *t*_(4)_ = 2.24, *p* = 0.044) was decreased in the shRNA-Gm16685-treated group compared to the control groups (Figure 4e). Thus, these data strongly suggest that the knockdown of Gm16685 promoted the polarization of macrophages to the M2 phenotype.

### 3.5. Further Study on the Mechanism of Action of Gm16685

**Figure 5:**
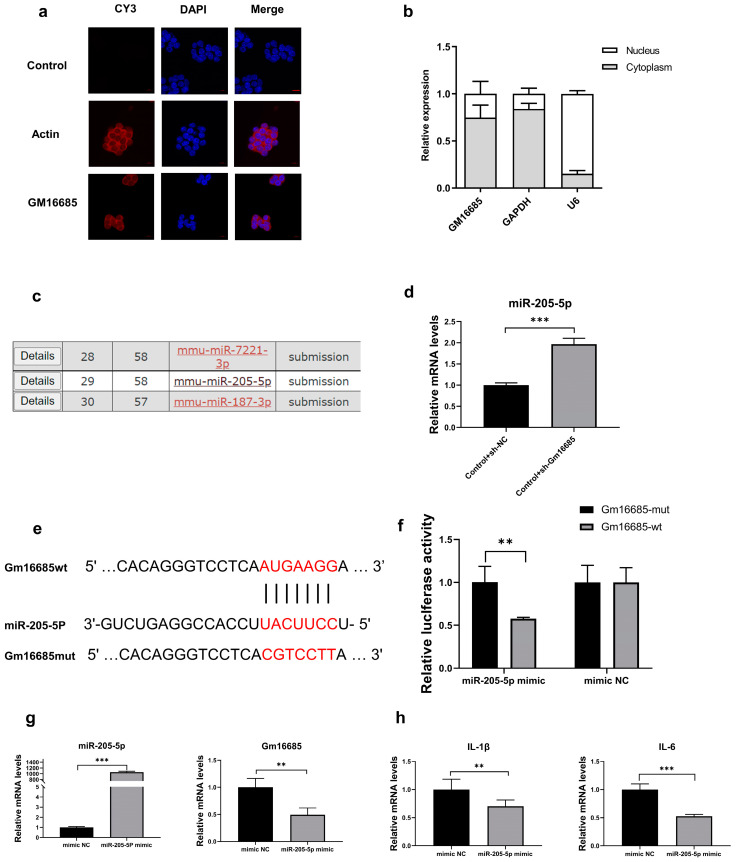
Further study on the mechanism of action of Gm16685: (**a**) Representative images of RNA fluorescence *in situ* hybridization (FISH) present Gm16685 (in red) localization in the macrophage cytoplasm. Actin mRNA is used as a reference and is present in the cytoplasm, scale bar = 20 µm. (**b**) qRT-PCR analysis of Gm16685 abundance in the nuclear and cytoplasmic fractions of RAW264.7 cells. (**c**) miRDB predicted the miRNAs that might interact with Gm16685. (**d**) qRT-PCR analysis of miR-205-5p expression after knockdown of Gm16685 in RAW264.7 cells. (**e**) The sequence fragments of wild type (wt) and mutant type (mut) of Gm16685 contained the binding site of miR-205-5p or the mutation binding site of miR-205-5p. (**f**) A luciferase reporter assay was used to determine the interaction between Gm16685 and miR-205-5p. (**g**) The expression of Gm16685 after overexpression of miR-205-5p was measured by qRT-PCR. (**h**) The expression of proinflammatory cytokine mRNAs after overexpression of miR-205-5p was measured by qRT-PCR. Data are presented as the mean ± standard deviation, n = 3. ** *p* < 0.01, *** *p* < 0.001.

FISH revealed that Gm16685 was predominantly localized in the cytoplasm of normal cultured RAW264.7 cells. Actin, used as a positive control, was mainly located in the cytoplasm (Figure 5a). Moreover, the nucleocytoplasmic separation experiment showed consistent results with FISH analyses (Figure 5b). miRDB predicted the miRNA that might interact with Gm16685 (Figure 5c). Through qRT-PCR analyses, it was found that miR-205-5p was upregulated after Gm16685 was knocked down in RAW264.7 cells (*t*_(4)_ = 10.95, *p <* 0.001) (Figure 5d). The interaction between Gm16685 and miR-205-5p was confirmed by luciferase reporter assay (Figure 5e,f). After overexpression of miR-205-5p, the expression of Gm16685 was significantly downregulated (*t*_(4)_ = 4.24, *p* = 0.007) (Figure 5g). The expression of inflammatory factors was also downregulated (Figure 5h).

## 4. Discussion

The regulatory functions of lncRNAs associated with liver disease have garnered increasing attention [30]. Regarding the regulation of lncRNAs on the biological function of liver cells, previous studies have focused on the progression of liver fibrosis and liver cancer. For instance, silencing lnc-LFAR1 decreases TGFβ-induced hepatocyte apoptosis, impairs HSC activation *in vitro*, and alleviates liver fibrosis induced by CCL4 [31]. lnc-LFAR1 plays a crucial role in regulating the activation and pyroptosis of macrophages, thereby providing an underlying target against inflammation-related diseases, including hepatic fibrosis [32]. lnc-TLNC1 promotes hepatocellular carcinoma progression and metastasis through the TLNC1-TPR-p53 axis [33]. However, the role of lncRNAs in liver lesions caused by schistosomiasis has rarely been studied.

One of the most serious clinicopathological features is liver egg granuloma caused by egg deposition during schistosomiasis [34]. Many lncRNAs are differentially expressed in the liver of *S. japonicum*-infected mice [25]. Hence, we first detected the expression profile of lncRNAs in the liver samples of mice infected with *S. japonicum* at different time points using RNA-seq. Many important lncRNA molecules related to the pathogenesis and progression of egg granulomas of *S. japonicum* were identified. Among the 12 genes selected, the expression levels of 0610039H22Rik, 5830428M24Rik, NR028126, H19, MEG3, and Mirt were upregulated after 45 days of schistosomiasis infection, which is the same as the study by Xia et al. [25]. According to the literature, H19 was also significantly induced by bile acids in mouse cholangiocytes [35]. Gm16685, also known as NAIL, was significantly upregulated in the colitis site of patients with ulcerative colitis. It can directly regulate the initiation and progression of colitis [36]. In this study, qRT-PCR results showed that the expression level of Gm16685 was increased 6-fold and 61-fold after 24 and 45 days of infection, respectively. Combining the results of high-throughput sequencing and qRT-PCR experiments, we found that Gm16685 was highly expressed in the liver of mice 45 days after *S. japonicum* infection. The expression level of Gm16685 was highly correlated with NF-κB activity. Genistein attenuates schistosomiasis liver granulomas by inhibiting the activity of NF-κB [34]. Therefore, our studies suggested that Gm16685 may exert effects on the pathogenesis of hepatic disease in schistosomiasis.

Interestingly, the knockdown of Gm16685 in mice infected with *S. japonicum* reduced the symptoms of egg granulomas, suggesting that Gm16685 participated in the pathogenesis of hepatic disease in schistosomiasis. Granuloma is an organized aggregation of macrophages and other immune cells, and the granuloma response is characterized by macrophage activation and transformation [37]. M1 macrophages can induce a chronic inflammatory state, whereas M2 macrophages can reduce this state in diseased tissues [38]. Our results suggest that the knockdown of Gm16685 in mouse liver can reduce the expression of M1 macrophage-related indicators and increase the expression of M2 macrophage-related indicators after *S. japonicum* infection, thus indicating that knocking down lncRNA-Gm16685 can inhibit M1 macrophage polarization and promote M2 macrophage polarization.

To further study the effects of Gm16685 on macrophages, hepatocytes, hepatic stellate cells, and macrophages were isolated from mouse models infected with *S. japonicum*. Gm16685 was significantly upregulated in macrophages. We also found that LPS treatment promoted Gm16685 expression in mouse macrophage RAW264.7 cells. The findings showed the proinflammatory effects of Gm16685 in macrophages. Meanwhile, in mouse macrophage lines, Gm16685 knockdown was consistent with the changing trend in tissues, with the downregulation of proinflammatory cytokines and upregulation of anti-inflammatory cytokines.

To date, a series of studies proved that subcellular localization of lncRNAs was critical to their function [39,40,41]. lncRNAs located in the cytoplasm can interfere with the posttranslational modification of the protein, serve as bait for miRNA, or regulate the translation, stabilization, and degradation of mRNA [17]. In our study, FISH and nucleocytoplasmic separation experiments revealed that Gm16685 was predominantly localized in the cytoplasm of normal cultured RAW264.7 cells. Thus, we performed a preliminary study on the mechanism of action of Gm16685. After the knockdown of Gm16685, the expression of miRNA-205-5p was upregulated. Previous research reported that the function of miRNA-205-5p was related to inflammation, and overexpression of miRNA-205-5p could inhibit inflammation [42]. Therefore, Gm16685 could repress conversion by acting as an miRNA-205-5p sponge.

## 5. Conclusions

This study established an expression profile of lncRNAs in the livers of mice with schistosomiasis at different time points. Notably, lncRNA-Gm16685 expression was upregulated in the livers of schistosomiasis mice. Gm16685 knockdown promoted M2 macrophage polarization and alleviated *S. japonicum*-induced hepatic granulomas. Additionally, Gm16685 promoted M1 macrophage polarization by regulating miR-205-5p. Thus, our study may provide a new target for schistosomiasis japonica treatment.

## Data Availability

The datasets generated and/or analyzed during the current study are available from the corresponding author on reasonable request.

## Supplementary Materials

The following supporting information can be downloaded at https://www.mdpi.com/article/10.3390/microorganisms11030796/s1, Table S1: Primer sequences of genes.

## Sample Availability

Samples are available from the authors.
