# Supplementary material for: Knocking Down Gm16685 Decreases Liver Granuloma in Murine Schistosomiasis Japonica
Source: Microorganisms. 2023 Mar 21;11(3):796. doi: 10.3390/microorganisms11030796 (PMC10058064; doi:10.3390/microorganisms11030796)
Supplement: Supplementary file 1 [file microorganisms-11-00796-s001.zip › microorganisms-2253621-supplementary.pdf]

Table S1. Primer sequences of genes.

	Primer	Sequence (5′-3′)
Actin	Forward primer	AGAGGGAAATCGTGCGTGAC
	Reverse primer	CAATAGTGATGACCTGGCCGT
MEG3	Forward primer	GGACTTCACGCACAACACGTT
	Reverse primer	GTCCCACGCAGGATTCCA
NR-028126	Forward primer	TGGGACCACTGAGCCAATTT
	Reverse primer	TACTGGCTTTCCAGGGGACT
Mirt	Forward primer	GAGTCCCCTCGTATCAGTGC
	Reverse primer	GCCGAGAATGACCCTGTACC
NR-110365	Forward primer	AGGCTGGGAGAGGGTATCAC
	Reverse primer	TGCAGCAGAAGAGTAGGGGT
5830428M24RIK	Forward primer	AGGATGTCAAGGGCAAAGGT
	Reverse primer	TTCGGAGCCTTGTTTCCACA
Cdkn1B	Forward primer	TACTAGGAAGGTACAGGCGGG
	Reverse primer	AGGACACAGGGCAAACTTCT
AI662270	Forward primer	AACTACCTCTGCCGCCATTT
	Reverse primer	GGGGTTCTGCCTCTTGATGT
1700025B11Rik	Forward primer	GAGAGTGTCGTCATCCCACA
	Reverse primer	CCAGCGATGGAGCTCAAGAC
0610039H22Rik	Forward primer	AACCGCAACTGCATCCTTCA
	Reverse primer	TCCAAGGAGCACAATCCACA
F630028O10Rik	Forward primer	TCCCATCCAAGACAAAGGCAC
	Reverse primer	TCCAGTTCACACCTTTCAGACC
Gm16685	Forward primer	GGACCTTGTCAAGCACATTCCAG
	Reverse primer	GAAAGCCCTCTTTAAAGCACC
H19	Forward primer	GAAGGCGAGGATGACAGGTG
	Reverse primer	CGGATTCAAAGGCCCAGACA
IL-12A	Forward primer	CTGTGCCTTGGTAGCATCTATG
	Reverse primer	GCAGAGTCTCGCCATTATGATTC
CCL1	Forward primer	TGCCGTGTGGATACAGGATG
	Reverse primer	GTTGAGGCGCAGCTTTCTCTA
IL-4	Forward primer	CTCATGGAGCTGCAGAGACTCTT
	Reverse primer	CATTCATGGTGCAGCTTATCGA
IL-10	Forward primer	TGAAGACCCTCAGGATGCGG
	Reverse primer	AGAGCTCTGTCTAGGTCCTGG
IL-13	Forward primer	CCTGGCTCTTGCTTGCCTT
	Reverse primer	GGTCTTGTGTGATGTTGCTCA
TGFβ	Forward primer	ACAATTCTTGGCGTTACCTT
	Reverse primer	AGCCCTGTATTCCGTCTCC
FIZZ1	Forward primer	CCCTCCACTGTAACGAAGACTC
	Reverse primer	CACACCCAGTAGCAGTCATCC
IL-1β	Forward primer	CTGAACTCAACTGTGAAATGC
	Reverse primer	TGATGTGCTGCTGCGAGA
IL-6	Forward primer	ACACATGTTCTCTGGGAAATCGT
	Reverse primer	AAGTGCATCATCGTTGTTCATACA
CD86	Forward primer	TGTTTCCGTGGAGACGCAAG
	Reverse primer	TTGAGCCTTTGTAAATGGGCA
CD80	Forward primer	ACCCCCAACATAACTGAGTCT
	Reverse primer	TTCCAACCAAGAGAAGCGAGG
U6	Forward primer	AACGCTTCACGAATTTGCGT

	Reverse primer	CTCGCTTCGGCAGCACA
miR-205-5p	Forward primer	CCTCCTTCATTCCACCGG
	Reverse primer	CCAGTGCAGGGTCCGAGGTA
